# Methods Used to Assess the 3D Accuracy of Dental Implant Positions in Computer-Guided Implant Placement: A Review

**DOI:** 10.3390/jcm8010054

**Published:** 2019-01-07

**Authors:** Se-Wook Pyo, Young-Jun Lim, Ki-Tae Koo, Jungwon Lee

**Affiliations:** 1 Department of Dentistry, Ajou University School of Medicine, Suwon 16499, Korea; pyosewook@hanmail.net; 2 Department of Prosthodontics and Dental Research Institute, School of Dentistry, Seoul National University, Seoul 03080, Korea; 3 Department of Periodontology and Dental Research Institute, School of Dentistry, Seoul National University, Seoul 03080, Korea; periokoo@snu.ac.kr; 4 Department of Periodontics, One-Stop Specialty Center, Seoul National University, Dental Hospital, Seoul 03080, Korea; jungwonlee.snudh@gmail.com

**Keywords:** dental implants, accuracy, computer-guided surgery, implant position, displacement measurement

## Abstract

The purpose of this review is to examine various assessment methods in order to compare the accuracy between the virtually planned and clinically achieved implant positions. In this review, comparison methods using pre- and post-operative computed topography (CT) data and digital impressions for definitive prosthesis will be described. The method for the displacement and strain for quantification of the error will also be explored. The difference between the planned and the actual implant placement position in guided implant surgery is expressed as an error. Assessing the accuracy of implant-guided surgery can play an important role as positive feedback in order to reduce errors. All of the assessment methods have their own inevitable errors and require careful interpretation in evaluation.

## 1. Introduction

Successful dental implant placement depends not only on osseointegration, but also on the function and esthetics of the final prosthesis [1,2]. Implant placement taking into consideration the appropriate angulation and position in relation to the adjacent teeth and underlying bone, or “prosthetically-driven placement”, is quickly gaining popularity.

Guided implant surgery allows for accurate, safe, and predictable implant placement [3]. Conventional implant planning is a model-based workflow that begins with a preliminary impression and diagnostic wax-up on the plaster model [4,5]. Before the development of digital technology, a radiographic template had to be fabricated over duplicated casts of a diagnostic wax-up. In computed topography (CT) imaging with templates, radiographic templates outline the proposed ideal prosthetic outcome relative to the patient’s anatomic structures and topography. The radiographic template can then be manually modified to the desired surgical template.

With significant achievements accomplished in the field of computerized implant dentistry, two types of techniques are now available, the “static” application of surgical templates, and “dynamic” transfer of the selected implant position to the surgical area via a navigation system [6]. While the latter provides real-time visual guidance in various situations during surgery, the former guided method of surgery is less flexible in regard to changing the surgical plan amidst the surgery, as the information is only transmitted through the surgical template. However, static guided implant surgery is still actively performed in clinical settings, because it does not require additional expensive pieces of equipment and complicated software. In addition, there are no time and space limitations. Despite such advantages, technical errors can cause serious problems in clinical applications [7].

The most significant problem in guided implant surgery is “deviation” between the planned and actual implant placement position. A number of factors may contribute to these inaccuracies. The possible causes for errors include spatial resolution problems in CT, merging techniques in CT, and scan data, errors in template manufacturing, inadequate stability of the surgical template, drilling errors from the clearance between the sleeve and the drill, as well as other factors, such as soft tissue thickness, patient movement, and the types of software used [8]. Therefore, clinical evaluation of the accuracy is essential to determine whether the inaccuracies of guided surgery are clinically acceptable.

Assessments of the accuracy of implant positions can be carried out at the preclinical stage using a resin model [9] or cadaver [10] in phantom studies, or they can be performed on the patient at the clinical stage (in vivo). It should be noted that in vitro experiments may be advantageous in identifying the causes of error due to the relatively large number of controllable variables. However, “deviations” may appear more inconsequential than they actually are in surgical settings.

In order to evaluate the accuracy of a guided implant surgery system, both the planned and actual positions of the implant are required. Recently, fully digital and model-free implant planning has become possible, and this digital approach begins with the acquisition of cone beam computed topography (CBCT) imaging and intraoral scanning data. A virtual model of the patient is created by superimposing the DICOM (Digital Imaging and Communications in Medicine) and STL (Standard Triangulated Language) files, allowing for a detailed visualization of the remaining dentition, surrounding intraoral soft tissue, and underlying bone structure. In the software, the implant fixtures are selected, and the drilling protocol is planned with respect to the final restoration and bone anatomy. A surgical template is then designed and fabricated.

The purpose of this review is to examine various assessment methods so as to compare the accuracy between the virtually planned and clinically achieved implant positions. In this review, the methods for determining the placed implant position and comparing the pre- and post-operative data will be described. In addition, the method of quantifying the errors associated with the displacement and strain are also explored.

## 2. Assessment Methods of 3D Computer-Guided Implant Placement

There are several assessment methods of 3D computer-guided implant placement. The position of the actually placed implant can be acquired by two methods. The first is by using postoperative CT images directly from a patient after implant surgery; the second method is indirectly estimating the placed position from the impression copings [11] or scan abutment [12] connected to the corresponding inserted implants. With the obtained information on the actual position of the placed implant, measuring the amount of displacement using different reference points is commonly used to quantify the difference between the planned and placed position [13,14]. Another method is to quantify the degree of displacement by assessing the strain that is applied when connecting a specially designed superstructure to a fixture analogue made from an acrylic resin model [15,16].

### 2.1. Direct Method (Pre- and Post-Operative CT Comparison)

In the direct method, the CT image is taken before and after the implant surgery, in order to radiographically confirm the three-dimensional position of the implants. The advantage of this method is that it can confirm the position of the implant immediately after the placement. However, the artifacts created from the material of the implant (titanium) can cause ambiguity of the radiographic outline of the implant. This ambiguity generates the overall enlargement of the implant contour, which results in a confirmation of the precise position only through estimation. Problems in the image precision and sharpness can be improved if the newly developing metal artifact reduction method (MAR) is introduced [17].

To accurately compare pre- and post-operative CT images, superimposition of the images using identical reference points is required. Using identical machines and superimposing with at least three fixed reference points are minimally required for an accurate CT comparison. In the most automatically superimposing software, multi-modality image registration is used to maximize the mutual information of two images [18].

In partially edentulous patients, tooth structures, which are relatively easy to identify, are used as reference points. Three different teeth, usually one anterior and two posterior, are selected for reference. For more accurate superimposition, pre- and post-operative CT scans can be performed using radiographic indices with three radiopaque markers, which can then be used as reference points for three-dimensional overlap. Such radiographic equipment is most often utilized for fully edentulous patients whose anatomical structures are not visible in the CT images. After CT images are superimposed, the scan information is used to create virtual three-dimensional models whose relative positions can be compared through 3D modeling analysis or CT image replacing analysis.

#### 2.1.1. 3D Modeling Analysis

**Figure 1:**
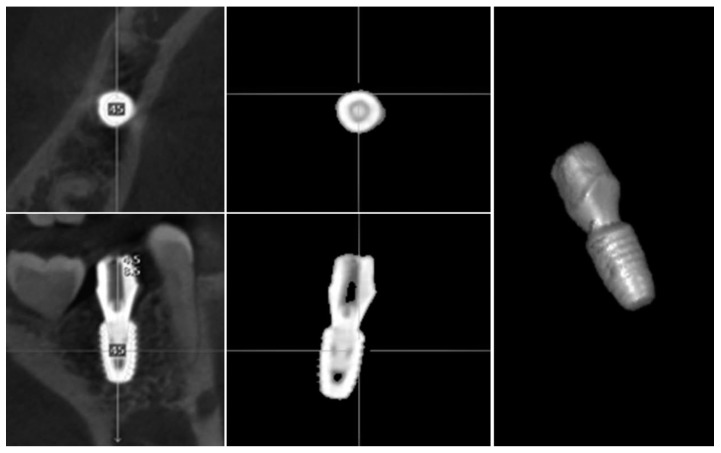
A portion of implant on the post-operative computed topography (CT) is separated. 3D modeling analysis confirms the actual position by directly reproducing the shape of the implant.

Three-dimensional modeling analysis constructs a virtual model from CT data and determines the position from the extracted implant portion (Figure 1). In three-dimensional modeling analysis, the implant portion of the CT image can be easily extracted, because its constituting material has a higher density compared to bone. However, artificial image artifacts prevent the exact extraction of the outline, and the resolution of the image can further affect the accuracy. The image cannot be used when the clarity is impaired by patient movement. Consequently, the virtual 3D model of a planned implant is the original library model with clear outlines, but the model of a placed implant has unclear outlines, making the comparison between these two models difficult.

#### 2.1.2. Image Replacing Analysis

**Figure 2:**
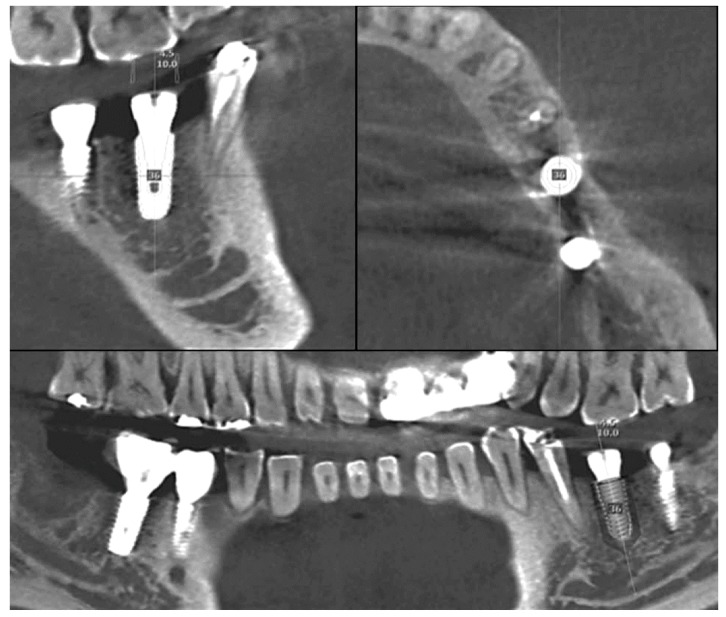
A library image of implant fixture is loaded on the post-operative CT. By this image replacing analysis, the implant placed position is determined.

Image replacing analysis substitutes implant the CT image with a cross-sectional CT image from the original implant library (Figure 2). Analysis is only possible when the spacing and direction of both of the CT image sections are matched. By overlapping the two images, the actually placed position of the implant can be determined and compared with the planned position.

### 2.2. Indirect Method (Pre- and Post-Operative Model Comparison)

An indirect method determines the implant position by taking an impression or by scanning through the impression coping or scan body connected to the implant. The impression coping is used to take an analog impression with silicone materials, while the scan body is used to take a digital impression with an intraoral scanner. This method uses the same principle as the final impression procedure for the definitive implant prosthesis fabrication. Therefore, the patient does not need to take an additional CT radiograph after implant surgery. However, if the impression coping or scan body is not correctly connected to the implant, there may be a critical error in the implant position. The impression coping or scan body can be connected immediately after the implant surgery or at the final impression stage, after the surgical site completely heals.

#### 2.2.1. Cast Analysis

**Figure 3:**
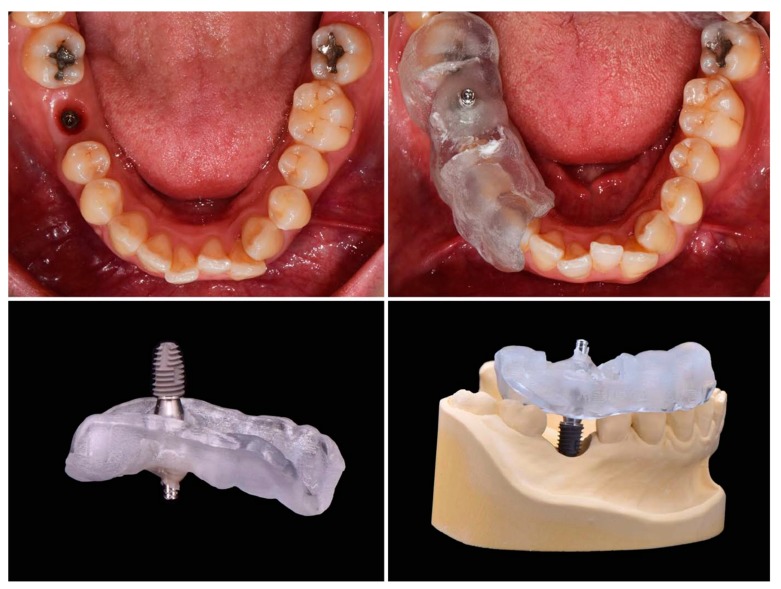
If the insertion torque is stable after implant surgery, the implant placed position can be determined using a surgical template immediately. The surgical template should be able to hold the impression coping connected to the placed implant.

The implant final impression procedure is performed after osseointegration. This allows for the determination of the position of the implant in the model. In general, the implant final model is fabricated with the implant analog replicating the internal structure of implant fixture. By connecting the scan body to the implant analog and scanning the final model, the implant placed position can be digitally verified. It is also possible to determine the implant position with a micro CT using an actual implant fixture instead of the implant analog. If initial stability is achieved during implant placement, impression coping can be connected immediately to the fixture, with caution, and also firmly fixed to the surgical template with a pattern resin or a light-cured resin. Then, the impression coping attached to the template should be removed from the fixture. Similarly, an implant fixture of the same size is connected to the impression coping. This template–coping–fixture complex can predict the position of the actual implant (Figure 3). It could be located on the preoperative gypsum model with the implant portion removed, and the implant fixture should be fixed again by the gypsum. In this way, the postoperative model is acquired and a cast analysis could be done. If there is no preoperative gypsum model, or if this method is used in fully edentulous patients, the additional CT radiographs could be used to superimpose with a preoperative CT. However, this method is not recommended because of incomplete superimposition and images even with more than three radiopaque markers on the template.

#### 2.2.2. Scan Analysis

**Figure 4:**
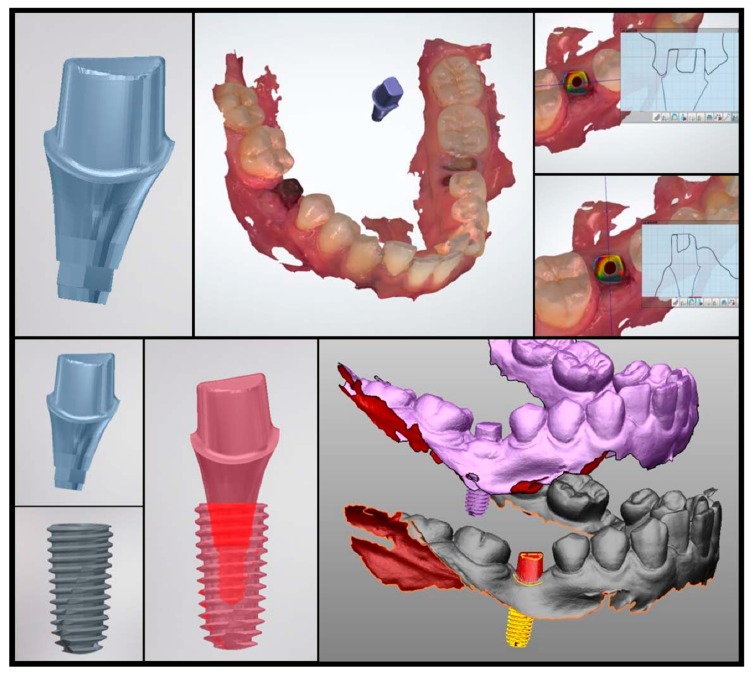
A custom abutment prefabricated to the planned position of the implant can be used as the scan body. By connecting this scan body to the implant and performing an intraoral scan, the actually placed position of the implant can be indirectly confirmed.

Instead of impression coping, an implant scan body can be used to determine the placed position with an intraoral scanner. In this case, a gypsum model is replaced with a digital model. This method indirectly confirms the position of the actual implant by matching the scan body outline and the virtual connecting of implant fixture. First of all, the position of the scan body should be completely matched in both the library file and the intraoral scan data. As this procedure is performed manually, however, the implant position can be altered significantly, even with a minimal error. Once the location of the scan body is determined, the implant position can be identified by a library image that virtually combines the implant fixture and scan body. Figure 4 shows the results of comparing the preoperative and postoperative scan copings on the patients who received implant guide surgery at Seoul National University Dental Hospital. Intraoral scanner (Trios3^®^, 3 Shape, Copenhagen, Denmark) and Implant studio™ (3 Shape, Copenhagen, Denmark) were used for analysis (Figure 4). It is also possible to compare it with the surrounding anatomical structure by superimposing the CT data before surgery. The indirect method (impression taking) has an additional step of connecting and removing the impression coping or scan body, therefore the possibility of an error is inevitable. In 2013, Platzer et al. [11] sandblasted a healing abutment that was designed to prevent rotation in pre-operative and post-operative implant, and connected it with 25 Ncm force, using the abutment as a scan body by digitizing it with a laser scanner. In the author’s opinion, it can be better to use a fixture mounter when placing an implant and obtaining the implant’s position information by intraoral scanner using the mounter as the scan body. This technique would be able to reduce the error.

## 3. Superimposition of the Planned and the Placed Implant Positions

To evaluate the error, the planned and the placed implant positions should be analyzed in the same space. In other words, two pieces of three-dimensional information need to be superimposed on a single identical plane. Superimposing two planes requires three or more different reference points that are not in a straight line. In partial edentulous, two pieces of data can be superimposed by reference points, such as three distinct teeth. In the complete edentulous, however, special reference points are necessary for superimposition.

CT data (anatomic information) and scan data (prosthetic information) should be merged in the implant planning stage. Therefore, the planned position can be described using either a CT image or scan image. As previously mentioned, the actual position of the placed implant can be confirmed directly by CT or indirectly by impression. As a result, the type of planned position data should be decided according to the method of determining the placed position for comparison.

**Figure 5:**
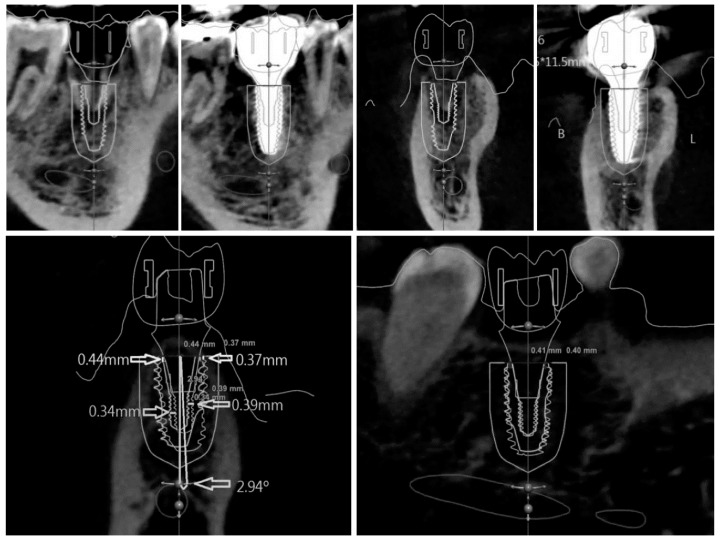
Displacement measurement by direct method. The planned position and the placed position are superimposed.

In Figure 5, information on the planned position was extracted from CT image, because the actual implant position was confirmed by “image replacing analysis” using CT. The reference point and the background data for comparison are all in the CT. Therefore, the accuracy of the superimposition is determined by the resolution of the CT, which consists of a DICOM file.

**Figure 6:**
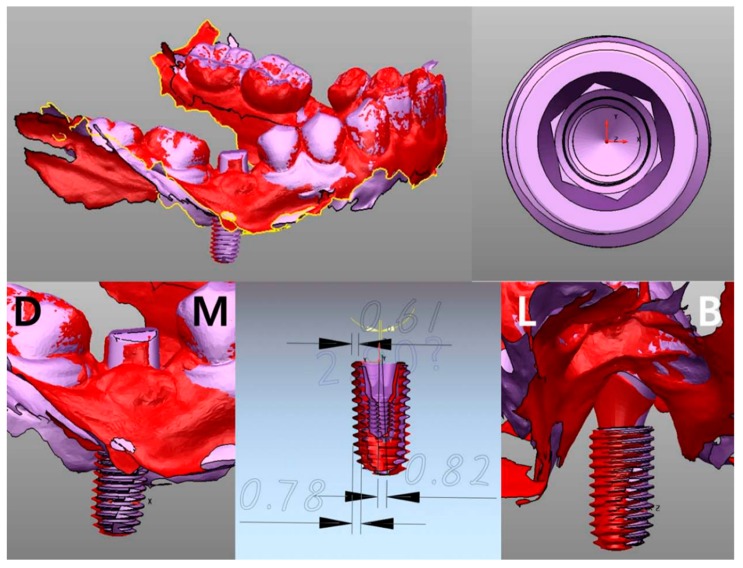
Displacement measurement by indirect method. The planned position (purple) and the placed position (red) are superimposed.

In a similar way, as the actual placed position of implant in Figure 6 was confirmed by the “scan analysis method” using the principle of impression taking, the planned position was loaded by the scan image (Figure 6). The reference point and the background data for comparison are all in the scan data. Consequently, the accuracy of the superposition is determined by the resolution of the intraoral scanner, which consists of a STL file.

The latter may be more advantageous for superimposition, because the narrow-ranged scan data that covers only the teeth has better defined borders than the CT data covering a relatively wide range. However, this method results in the indirect confirmation of the implant position through impression coping, which creates an additional possibility of errors.

## 4. Quantification of the Errors

### 4.1. Displacement Measurement

**Figure 7:**
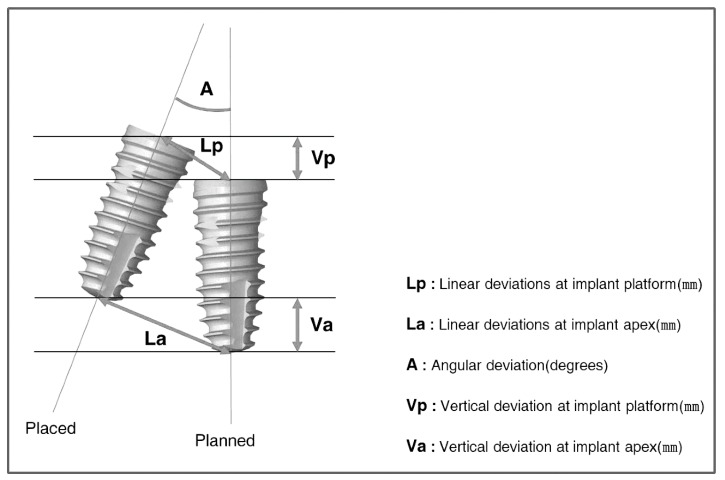
These parameters used to displacement measuring in general. “Lp” and “La” can each be reported by one distance, or by two individual vectors (with a horizontal and a vertical distance).

If the planned and placed positions are superimposed using the direct or indirect method, the amount of error can be analyzed by measuring the displacement. In order to specify the displacement, a standard parameter is needed to calculate/quantify the position of the implant. The following parameters are used for displacement measuring in general (Figure 7): (1) linear deviations at the implant platform, (2) linear deviations at the implant apex, (3) angular deviation, and (4) vertical deviation in height or depth. The parameters can each be reported as distance, or as two individual vectors (with a horizontal and a vertical distance) [19].

As the implants are roughly columnar, each central axis can be set as the reference. In a three-dimensional space, the relations of two axes are parallel or twisted to each other, or combined to meet at one point. With the exception of some specific cases, such as being parallel or twisted, two axes in a space meet at one point, to be brought into one plane. Two axes in the same plane can each be defined as the slope of line, and the line segment is composed of the entry and end point. Therefore, the relationship between two implants can be expressed by the intersection angle and the length of each point.

According to the review article by Tahmaseb et al. [20], 24 studies of implant guided surgery showed the deviation at an entry point to be an average of 1.12 mm and maximum value of 4.5 mm, the deviation at the apex point showed an average of 1.39 mm and maximum value of 7.1 mm, and the deviation of axis showed an average of 3.89° and maximum value of 21.16°.

### 4.2. Strain Measurement

In order to evaluate the fine error, Tahmaseb et al. [15,16] suggested a method to quantify the strain (force) on each implant, using strain gauge measurements. In this research, six implants were inserted using the drill guide attached to the anchor pins. After the final impression of the implants, a milled titanium structure was fabricated. Four strain gauges were attached along the long axis of each cylinder of superstructures at 90° angles to each other. Each frame was measured by a three dimensional tension-measurement method utilizing strain gauges. This method has the advantage of being able to express an indistinguishable error numerically. Although there are limitations to strain gauges, such as the location, number, and area of application, its role should not be overlooked. In addition, this method is not possible with a virtual model of a digital type, so a dental gypsum model of analog type must be fabricated.

## 5. Conclusions

Implant guide surgery is prone to errors because it is performed by many steps. Therefore, it is essential to evaluate the accuracy of implant placement in each patient. Evaluating the accuracy of implant guided surgery can be divided into the following three stages: (1) the assessment of the position of the actually placed implant, (2) the superimposition of the planned and the placed positions, and (3) the quantification of the errors.

The position of the actually placed implant can be confirmed by the direct method using post-operative CT and by the indirect method using an impression-taking procedure. In the direct method, the implant position can be determined by either 3D modeling analysis, which analyzes the CT-extracted implant image, or the image substitution analysis, which applies the position of the original implant library image to CT image. In the indirect method, the position of the implant can be determined through an indicator connected to an implant. The indirect method is classified as a model analysis method, in which the impression coping is attached to a surgical guide or an impression material, and a scan analysis method, in which a scan body is connected to an implant, so as to estimate a position using an intra-oral scanner.

**Figure 8:**
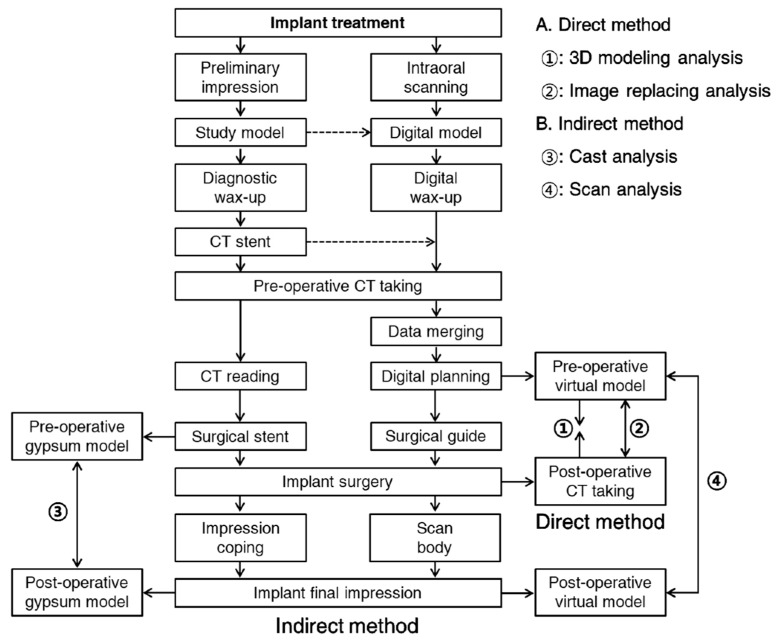
The process flow of the implant treatment and the principles of direct and indirect methods.

Figure 8 is a schematic diagram of the flow of implant guided surgery. This diagram shows which step corresponds to each comparison method.

The step of superimposition is determined according to the method of confirming the placed implant position, and three or more reference points are required for both the CT and scan data. However, the superimposing scan data is generally more accurate than the superimposing CT data. The quantification of error is performed by measuring the difference in the implant position, which is visible with the naked eye. For the finer errors, measuring the strain between the implant and superstructure can be used for the quantification of error. The direct method using post-operative CT to evaluate the accuracy of the guided surgery can intuitively provide the anatomical relationship and immediate surgery results directly to patients, but has the disadvantage of additional radiation exposure. Furthermore, errors may occur in the superimposition step, depending on the resolution of the CT image.

Although the indirect method using an impression-taking mechanism is safe from radiation exposure and is easy to superimpose, drawbacks exist, in that the impression coping or scan body can only be connected to the implant when the initial fixation or osseointegration is appropriate. The connecting procedures are also error prone as well. For the most conclusive evaluation of accuracy, cross-validation using the direct method and indirect method is recommended.

## 6. Future Directions

Implant guided surgery begins by assessing the accuracy of the actually placed implant. Within the current technical limits, it is better to use both the direct and indirect methods to define the position of the placed implants. However, it is expected that an optimization algorithm that can automatically recognize and exactly extract the border of the implant from the CT image will be developed in near future. This algorithm requires the help of artificial intelligence, because the experience using the direct method and indirect method must continuously be accumulated. Not only “extracting” the border of the implant, but also “superimposing” the pre- and post-operative information can also be performed automatically by artificial intelligence.

Acquiring the information of the implant position is equivalent to taking the final impression of the implant. Post-operative CT, therefore, can be used as both the final impression and accuracy evaluation of the placed implant. As a result, the development of the extraction and superimposition process by artificial intelligence will eliminate the implant impression-taking process. The absence of an impression procedure leads to the automation of the dental prosthesis manufacturing process, and the efforts of dental technicians are decreased. In the next generation, a technique utilizing the real time information of implant position will be developed. This implies that implant guided surgery will be replaced by implant navigation surgery. Implant navigation surgery will lead to implant robotic surgery, and the dentist’s efforts will be reduced.

As technologies develop, the fundamentals become more important. Today, implant surgical techniques are of interest to dentists. In the future, however, the conceptual factors of implant treatment will be more important than the technical factors. Therefore, clinicians have to know the criteria for what implants to use and where to place them.
